# Separation of Partial Discharge Sources Measured in the High-Frequency Range with HFCT Sensors Using PRPD-*t_eff_* Patterns

**DOI:** 10.3390/s20020382

**Published:** 2020-01-09

**Authors:** Ricardo Albarracín-Sánchez, Fernando Álvarez-Gómez, Carlos A. Vera-Romero, Johnatan M. Rodríguez-Serna

**Affiliations:** Department of Electrical and Electronic Engineering, Automatic Control and Applied Physics, School of Industrial Design and Engineering (ETSIDI), Universidad Politécnica de Madrid (UPM), Ronda de Valencia 3, 28012 Madrid, Spain; fernando.alvarez@upm.es (F.Á.-G.); ca.vera@upm.es (C.A.V.-R.); johnatan.rodriguez.serna@alumnos.upm.es (J.M.R.-S.)

**Keywords:** partial discharges, insulation condition, on-line PD measurements, wideband PD measurements, effective time, PRPD-time tool, PRPD-*t_eff_* pattern, noise rejection, pulse source separation

## Abstract

During the last two decades, on-line partial discharge (PD) measurements have been proven as a very efficient test to evaluate the insulation condition of high-voltage (HV) installations in service. Among the different PD-measuring techniques, the non-conventional electromagnetic methods are the most used due to their effectiveness and versatility. However, there are two main difficulties to overcome in on-line PD measurements when these methods are applied: the ambient electric noise and the simultaneous presence of various types of PD or pulse-shaped signals in the HV facility to be evaluated. A practical and effective method is presented to separate and identify PD sources acting simultaneously in HV systems under test. This method enables testers to carry out a first accurate diagnosis of the installation while performing the measurements in situ with non-invasive high-frequency current transformers (HFCT) used as sensors. The data acquisition in real-time reduces the time of postprocessing by an expert. This method was implemented in a Matlab application named PRPD-time tool, which consists of the analysis of the Phase-Resolved Partial Discharge (PRPD) pattern in combination with two types of interactive graphic representations. These graphical depictions are obtained including a feature parameter, effective time (*t_eff_*), related to the duration of single measured pulses as a third axis incorporated in a classical PRPD representation, named the PRPD-*t_eff_* pattern. The resulting interactive diagrams are complementary and allow the pulse source separation of pulses and clustering. The effectiveness of the proposed method and the developed Matlab application for separating PD sources is demonstrated with a practical laboratory experiment where various PD sources and pulse-type noise interferences were simultaneously measured.

## 1. Introduction

Developments in signal processing techniques achieved in recent years have positioned on-line partial discharges (PD) tests measuring with high-frequency current transformer (HFCT) sensors as an effective method for evaluation of the insulation condition of high-voltage (HV) electrical equipment such as power transformers [1]. The post-processing of the measured signals allows extraction of additional information and inference of the real insulation condition under specific operational situations [2].

A practical and efficient computational tool named the phase-resolved partial discharge (PRPD)-time tool was developed in order to assist in the insulation diagnosis procedure. The PRPD-time tool is a PD analysis tool that makes possible the supervised source-pulse separation process using a powerful and interactive graphical interface. A source pulse separation and identification process allows the determination of the specific PD or noise sources present simultaneously in HV installations and is commonly implemented in three stages [3]:

1. Filtering and signal denoising.
2. Feature extraction.
3. Individual patterns extraction.

The PRPD-time tool allows pulse separation using the effective time as a feature parameter [4] and bivariate distributions (phase *φ*—pulses amplitude *mV*—effective time *t_eff_*). The effective time of different pulses in the ultra-high-frequency (UHF) range in combination with power ratio analysis have been previously used with success for the separation of several PD sources [4].

The Matlab-developed application introduces for the first time the use of the effective time parameter *t_eff_* as a third dimension of classical PRPD patterns [5], allowing the separation of PD sources and noise signals. The acquisitions have been performed in the high frequency (HF) range using non-invasive HFCT sensors. In addition, PRPD-time tool allows the visualization of the resulting individual univariate time-resolved patterns (time *t*—pulses amplitude mV), which enables further information extraction such as the required in the analysis of the aging process of the individual insulation defects [5].

An adequate diagnosis based on PD measurements requires an accurate separation procedure in which pulses from different sources, including noise, are categorized and grouped depending on features and properties of their pulse shape [6]. Feature parameters for pulse separation can be extracted from its pulse shape in the time domain or, after transformations, in the frequency domain. Indeed, a combination of time and frequency domain analysis allow extraction of effective information for improving pulses separation [3]. The separation process is implemented in a feature map where the feature parameters are pairwise represented [7]. Once the pulses are effectively separated, PD sources can be identified using methods such as the PRPD patterns [8].

### 1.1. Time–Frequency Maps

In this approach, the equivalent time length and bandwidth of the pulses are the feature parameters. In this approach, it is considered that pulses from different sources have different pulse shapes. The equivalent time length and bandwidth are respectively calculated as [9]:

(1) $$T^{2}=\sum_{i=1}^{N}{\left(t_{i}-t_{0}\right)}^{2}s_{i}^{2}/\sum_{i=0}^{N}s_{i}^{2}$$

(2) $$BW^{2}=\sum_{i=0}^{N}f_{i}^{2}{\left|X_{i}\left(f_{i}\right)\right|}^{2}/\sum_{i=0}^{N}{\left|X_{i}\left(f_{i}\right)\right|}^{2}$$

where *N* is the number of samples from the measurement, *s_i_* is the sample measured at time *t_i_*, *t*_0_ is defined as time barycentre ($t_{0}=\sum_{i=1}^{K}t_{i}s_{i}^{2}/\sum_{i=0}^{K}s_{i}^{2}$) and *X_i_*(*f_i_*) are the frequency components of the PD signal calculated using the Fast Fourier Transform (FFT). Results are plotted on a 2D map for making the pulse source separation.

A similar procedure as previously presented, but not using the FFT, can be implemented using Haar and Walsh transforms, which use functions with a better fitting to typical PD pulses than sinusoidal functions in Fourier transforms [10]. On the other hand, for avoiding losses of information in the time domain of extracted features, wavelet transforms can be applied instead of Fourier transforms [11,12].

The time–frequency maps method has been extensively used indeed at High-Voltage Direct Current (HVDC) [13]. However, it is affected by settings such as sampling frequency, acquisition time, number of samples and vertical resolution of the acquisition. This affects the repeatability and generalization of conclusions [14].

In [15] the following feature parameter is also considered:

(3) $$f_{0}=\sum_{i=0}^{N}f_{i}{\left|X_{i}\left(f_{i}\right)\right|}^{2}/\sum_{i=0}^{N}{\left|X_{i}\left(f_{i}\right)\right|}^{2}$$

defined as the centre frequency of the signal. Pulses from different sources measured at UHF in a 500-kV substation were successfully separated using 3D (*T*^2^-*BW*^2^-*f*_0_) maps and fuzzy clustering.

In [16] a PD signal separation algorithm based on the cumulative energy in time and frequency domain, was presented. Four new feature parameters, calculated from the mathematical morphology gradient, which describes the variation characteristics of cumulative energy in time and frequency domain, were used and an improved density-based spatial clustering algorithm was implemented to discover clusters in the feature space and separate mixed signals.

### 1.2. Power and Energy Maps

Other methods use as feature parameter for separation, the power and energy of pulses, mathematically calculated after pulses were transformed into the frequency domain. The power distribution of signals in relevant frequency bands, in which there is higher dispersion in the accumulated power and are characteristic for different PD sources, allows separation of PD sources. Usually two frequency bands are considered and the power ratio (*PR*) in each one is calculated as follows if s(t) is the sampled PD pulse [17]:

(4) $$\%PRL=\frac{\sum_{f_{1L}}^{f_{2L}}{\left|\tilde{S}\left(f\right)\right|}^{2}}{\sum_{0}^{f_{t}}{\left|\tilde{S}\left(f\right)\right|}^{2}}\times 100\%$$

(5) $$\%PRH=\frac{\sum_{f_{1H}}^{f_{2H}}{\left|\tilde{S}\left(f\right)\right|}^{2}}{\sum_{0}^{f_{t}}{\left|\tilde{S}\left(f\right)\right|}^{2}}\times 100\%$$

where *PRL* is the power ratio in the low-frequency band, *PRH* is the power ratio in the high-frequency band, $\left|\tilde{S}\left(f\right)\right|$ is the fast Fourier transform magnitude, the low-frequency band is in the range [*f*_1*L*_,*f*_2*L*_], while the high-frequency band is in the range [*f*_1*H*_,*f*_2*H*_] and *f_t_* is the highest frequency value to be analysed.

*PRL* and *PRH* can be used as feature parameters in a univariate representation for separating different sources on a 2D map. An enhancement of this method was presented in [4] where a third axis is added to the *PR* map, for allowing the effective separation of sources with a similar power ratio. The third axis is the effective time that is defined as the duration of a rectangular pulse with the same peak as the squared signal whose integral gives the same energy as the original pulse [4].

The chromatic technique [18] is another method that uses the signal energy, its characteristic angular frequency (*ω_c_*) and the Root-Mean-Square (RMS) bandwidth (*B*) as feature parameters from the signal and its Fourier transform for generating 3D maps. Those maps allow separation of pulses from different sources such as corona, surface and internal PD measured using different inductive sensors [19,20].

In [21] a separation technique is proposed, which uses as feature parameters the current peak value, *I_peak_*; the PD charge amplitude, *Q* and the energy, *E_k_*. The current peak value is calculated using the HFCT signal, *v_k_* and *Q* is calculated as the integral over the time duration of the PD current pulse. The energy is calculated as:

(6) $$E_{k}=\frac{dT}{R}\sum_{1}^{N}{\left(v_{k}\right)}^{2}=\frac{dT}{R\cdot N}\sum_{1}^{N}{\left|\tilde{v_{k}}\right|}^{2}$$

where *R* is the input resistance of the acquisition system, *N* is the number of samples, $\tilde{v_{k}}$ is the Fast Fourier Transform (FFT) of $v_{k}$, *dT = 1/Fs*, *Fs* is the sampling frequency. The equality in Equation (6) makes use of Parseval’s theorem. This approach has been also used for PD clustering applications under unsupervised conditions [7] using an improved density peak clustering (DPC) method and pairwise representations from *I_peak_*, *Q* and *E_k_*.

In automated separation and identification methodologies, after feature maps are obtained, clustering algorithms or correlated calculations using reference pulses can be employed in order to perform the pulse sources separation [22]. Different clustering techniques and pattern recognition methodologies have been used such as fuzzy [9], support vector machine algorithms [23] and neural networks and extension neural networks [24]. On the other hand, in supervised sources separation tools, feature maps are plotted and experts can implement visually assisted procedures for executing the sources separation. After the separation is met, a PD identification is required which can be done using PRPD patterns [7].

Compared with the aforementioned separating methods, the developed PRPD-time tool allows a non-expert and low-computational cost procedure for PD source separation using the classical PRPD pattern and including a third axis corresponding to the effective time. The implementation of this processing application in Matlab is presented in Section 2. The laboratory experimental setup performed to check the effectiveness of the developed tool is shown in Section 3.1. In Section 3.2 the PD measurements obtained from the experimental setup are presented. The applicability of the PRPD-time processing tool is explained in Section 4, where the measured PD and noise sources are separated. Finally, Section 5 includes the conclusions obtained from this research.

## 2. PRPD-*t_eff_* Patterns

The classical PRPD patterns represent the amplitude or charge of the acquired pulses against the phase angle in which they occur. Thus, the pulses are seen super-imposed in one period of the test voltage. These patterns are widely used for the identification of PD sources, because each source features a different characteristic behaviour in the resulted image. Some expected patterns are [25]:

- Internal PD appears slightly before the zero crossings and in the increasing intervals of the test voltage.
- Surface PD appears in the increasing intervals of the test voltage and at the peak values. The PRPD pattern exhibits an asymmetry among positive and negative half-cycles.
- Corona PD pulses have a similar amplitude and the PRPD pattern exhibits great density about the maximum values of the test voltage. The amplitude can vary on each half-cycle (according with the type of corona).
- Electrical noise is usually not correlated with the voltage reference signal and depends on the type of the electrical noise.
- Pulses from IGBTs in the resonant system are synchronized with the test voltage and appear at its zero-crossings.

Therefore, PD pulses from the same source are positioned in defined regions when they are plotted on PRPD patterns. However, when several PD sources are simultaneously measured, their PD patterns together with the noise pulses can be overlapped. In these cases, the realization of accurate diagnosis is very difficult and sometimes even impossible. To solve this issue, the use of the proposed feature, called effective pulse width or effective time duration (*t_eff_*), is very useful. The implementation of this parameter, which is extracted from the measured pulses, enables the separation of the PD sources shown in the PRPD patterns using it as a third dimension.

### 2.1. Steps Used for Separating PD Sources Using the PRPD-t_eff_ Concept

**Figure 1:**
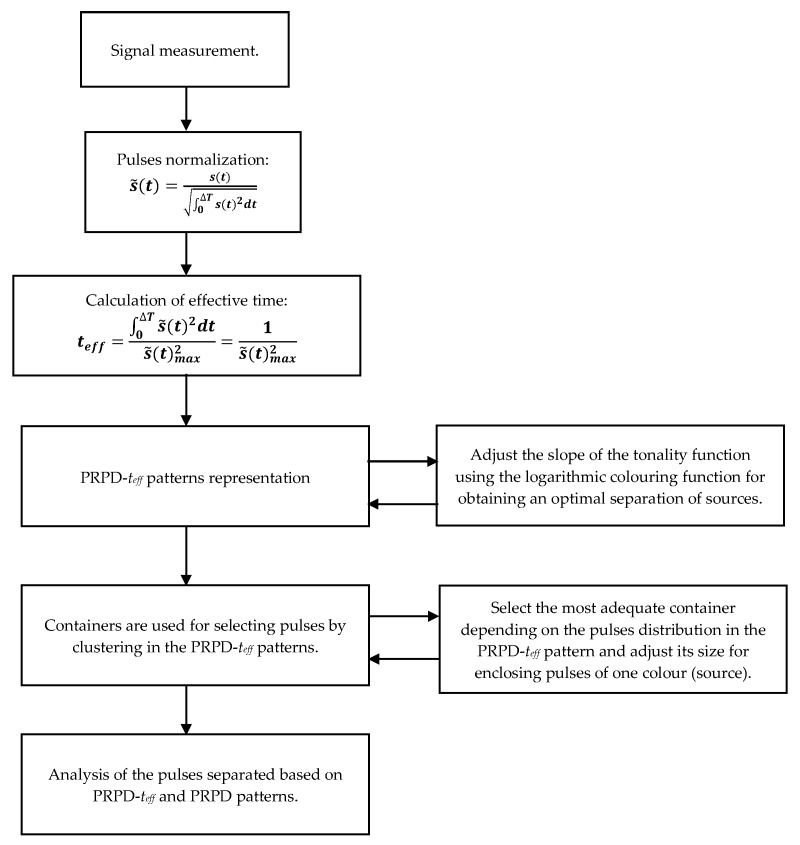
Flowchart used for separating PD sources using the PRPD-*t_eff_* concept.

The proposed method relies in the fact that pulses from different sources occupy specific zones in the PRPD-*t_eff_* pattern. The method is summarized in Figure 1 and implemented in the following steps:

1. A first denoising is required in order to filter background continuous noisy signals.
2. Each pulse is normalized in time domain, Section 2.2.
3. The effective time of each signal is calculated, Section 2.2.
4. PRPD-*t_eff_* patterns are plotted adjusting a logarithmic colouring function. The slope of the logarithmic function controlling the colour tonality, Section 2.3, can be manually adjusted for improving the separation.
5. Containers are used in the PRPD-*t_eff_* pattern for plotting classical PRPD patterns (pulse amplitude vs. phase angle) selecting sources of similar *t_eff_*.
6. Pulse sources are separated using the following two criteria:
  - Its location in the PRPD-*t_eff_* patterns.
  - Its PRPD pattern.

### 2.2. Signal Time Duration

The PRPD-*t_eff_* pattern is obtained by adding to the classical PRPD pattern, an axis corresponding to the effective time duration of each acquired pulse. To calculate this feature, in a first step the normalized pulse with time $\tilde{s}\left(t\right)$ is obtained:

(7) $$\tilde{s}\left(t\right)=\frac{s\left(t\right)}{\sqrt{\int_{0}^{\Delta T}s{\left(t\right)}^{2}dt}}$$

being Δ*T* (s) the time window in which each pulse s(t) is acquired.

The normalized pulse with time is used to calculate, in a second step, the effective pulse width *t_eff_* that represents the equivalent time length of each signal. This parameter is related to the width of a rectangular pulse with the same peak value as a squared signal whose integral has the same energy as the original pulse, thus both signals have the same area. The *t_eff_* (s) is expressed as follows [4]:

(8) $$t_{eff}=\frac{\int_{0}^{\Delta T}\tilde{s}{\left(t\right)}^{2}dt}{\tilde{s}{\left(t\right)}_{max}^{2}}=\frac{1}{\tilde{s}{\left(t\right)}_{max}^{2}}$$

where the numerator in Equation (8) is the integral of the signal for the whole acquisition window in which the event is registered Δ*t* (s), $\tilde{s}\left(t\right)$ is the normalized PD pulse and $\tilde{s}{\left(t\right)}_{max}$ is the maximum value of the PD or noise signal.

### 2.3. PRPD-Time Tool

The PRPD-time tool is programed based on the flowchart and steps shown in Section 2.1. The effective time of pulses is calculated and the PRPD-*t_eff_* pattern is depicted using it. Both tasks can be performed in real time (during the PD test) and with very low processing requirements. 

**Figure 2:**
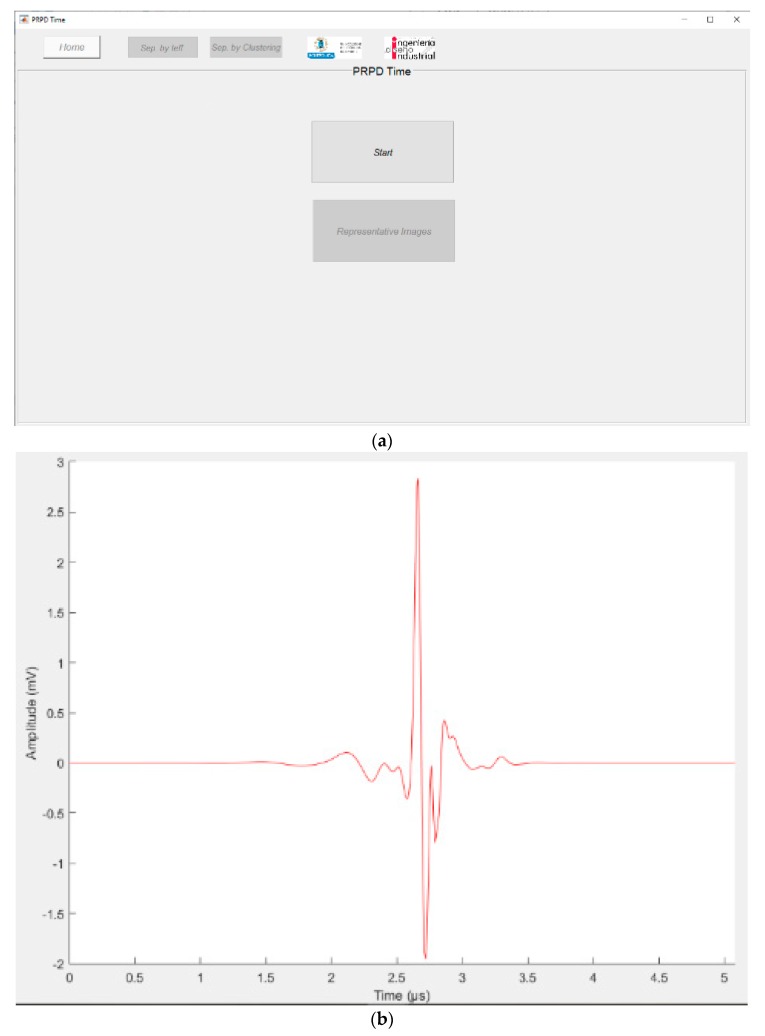
(**a**) Phase-resolved partial discharge (PRPD)-time tool, login window and (**b**) visualization of one of the pulses analysed.

Once the diagnosis application is initialized (Figure 2a), it is possible to analyse a file containing the measured pulses. Figure 2b shows as an example the wave shape of one signal acquired and processed with this tool. 

**Figure 3:**
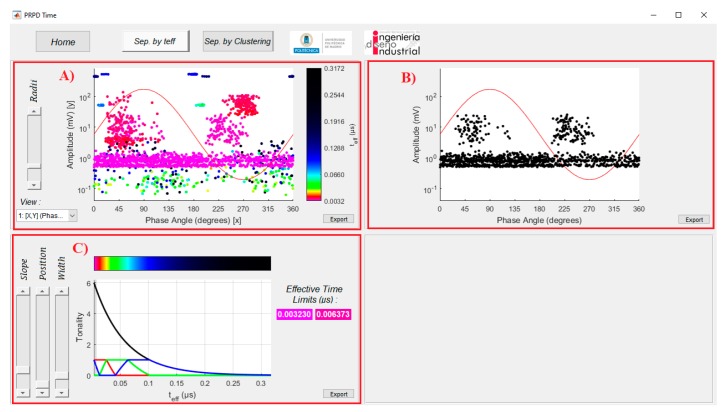
PD sources separation using the *t_eff_* parameter. (**A**) PRPD-*t_eff_* pattern; (**B**) classical PRPD pattern for the effective time range specified in (**C**); (**C**) controls for PD sources differentiation in PRPD-*t_eff_* patterns.

At the upper part of window in Figure 2a there are three tabs controlled by the buttons: *Home*, and the separation tools named *Sep. by teff* and *Sep. by Clustering*. The visualization related with *Sep. by teff* tab is shown in Figure 3. In this window, a first analysis can be performed considering the effective time parameter and using the sliders available in Figure 3C, in order to make a separation of pulses generated by different sources (Figure 3A). The slider *Position* controls the centre of a range of effective time to be covered in order to analyse a specific source, whose limits will be controlled with the value of the slider *Width*.

**Figure 4:**
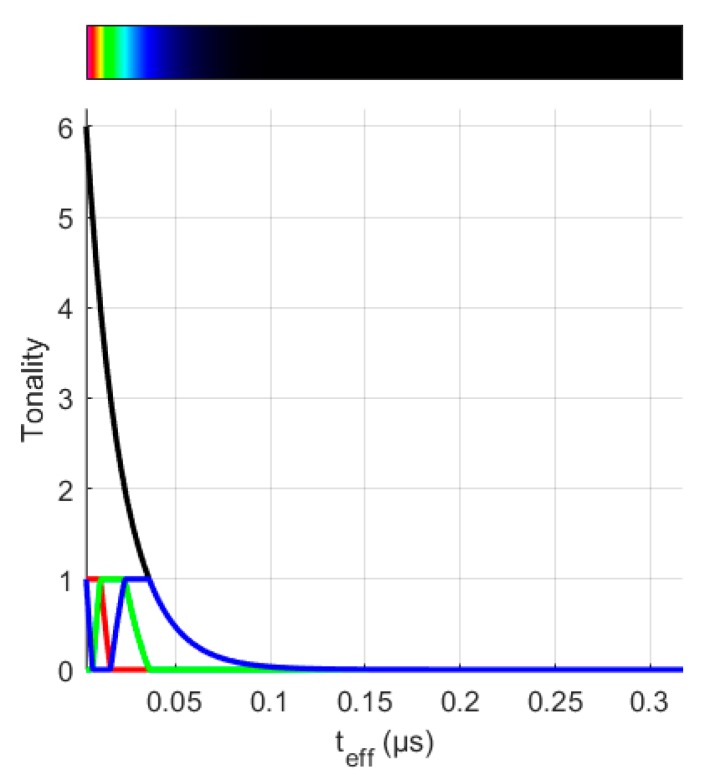
Colour and range selection of the effective time for representing the PRPD-*t_eff_* patterns.

However, taking into account that the PD duration is in the range of tens of nanoseconds to few microseconds [26], the variations of the feature parameter *t_eff_* among different PD sources is small. For this reason, in order to improve the visualization and ease the classification procedure, a function, which assigns a scale of colours to the pulses’ representation considering their effective time magnitude in a specific range, was implemented. The tones of the colours in the PRPD-*t_eff_* patterns is defined by a logarithmic function controlled by six states with different combinations for defining the RGB colour specification triplets in Matlab. Figure 4 shows a detail of the logarithmic definition of the tonality and the RGB triplets combination. The slider *Slope* modifies the curve that controls the transition between the six states, producing the differences in tones among the different PD sources. Figure 3B, shows the PRPD pattern for the selection made in Figure 3C.

**Figure 5:**
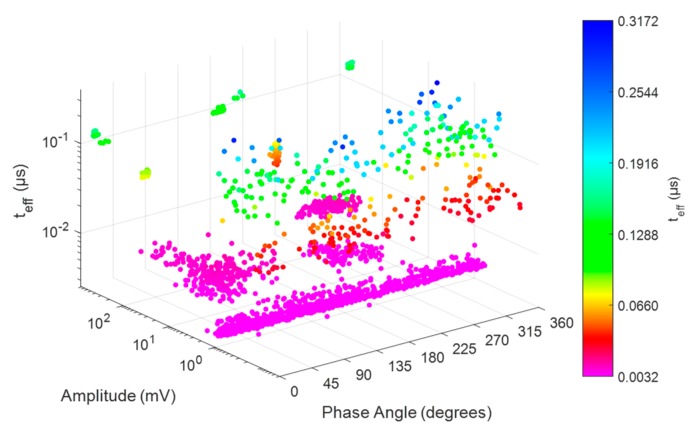
Example of PRPD-*t_eff_* pattern representation, isometric representation of phase angle-amplitude-*t_eff_*.

Once the range of colours for the effective time is selected, it is possible to represent the corresponding PRPD-*t_eff_* pattern for all pulses considered using an isometric representation of phase angle-amplitude- *t_eff_*, Figure 5, by selecting the option “Isometric” in the “View” list at the lower left-hand side of the Figure 3A.

A first rough separation is possible adjusting the *Slope*, *Width* and *Position* variables in the *Sep. by teff* button, Figure 3. 

**Figure 6:**
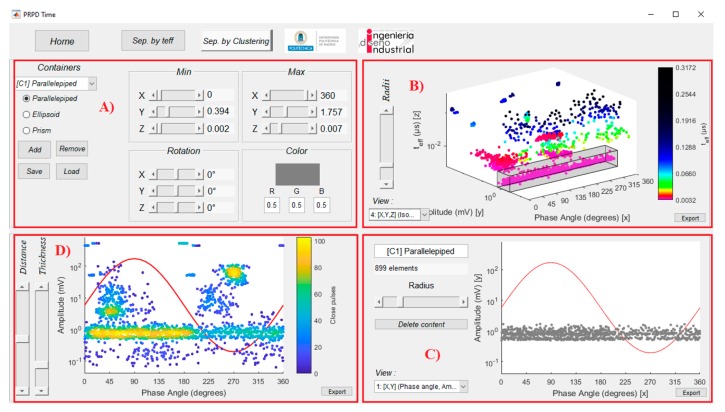
Graphical interface for pulses separation using the PRPD-*t_eff_* patterns. (**A**), Container definition: geometry, origin location and size; (**B**) container geometry plotted in the PRPD-*t_eff_* pattern; (**C**), pulse density pattern plot, distance and thickness controls and (**D**), PRPD pattern for pulses in container that allows the classification by clustering (PDCTC).

For further analysis and in order to complete the PD sources separation process, by pressing the button *Sep. by Clustering*, at the upper part of window in Figure 3, a new interface is activated, Figure 6A and the PD classification tool by clustering (PDCTC) is displayed, Figure 6B. The colours and tones in which pulses are represented in graphics of window in Figure 6, depend on the definition of tones given in the window related to the *Sep by teff* tab, Figure 3.

Using the cursors of the interface shown in Figure 6A the post-processing of the PRPD-*t_eff_* patterns under study is possible by selecting 3D geometrical shapes, called “containers”. The “containers” shape can be parallelepipeds, ellipsoids or prisms.

The separation of pulse sources is performed by selecting with the containers, the clusters formed in the PRPD-*t_eff_* visualization in Figure 6B, and analysing the corresponding individual PRPD pattern, which is automatically generated for pulses in that single cluster and shown in Figure 6C. These individual patterns can be associated to PD or noise sources. 

Once an individual PRPD pattern has been selected using the PDCTC, a density plot is shown in Figure 6D where it is possible to observe the concentration of pulses in the different regions of the PRPD pattern. In this way, the first rough separation presented in Figure 5, is improved upon and the individual sources of pulses are effectively separated using both tools, *Sep. by teff* and *Sep. by Clustering*.

## 3. Validation of the Analysis Tool

### 3.1. Experimental Setup 

The measurements performed for the validation of the proposed analysis tool were carried out in the HV laboratory LAT-UPM of the Universidad Politécnica de Madrid.

An experimental setup was assembled with various PD defects characteristic of insulated cables. In the test performed an HFCT sensor and a commercial PD measuring instrument [27] were used. 

#### 3.1.1. PD and Noise Pulse Sources

PD sources are briefly described as follows. Internal discharges were generated due to a cavity at the end of the semi-conductive layer in a cable termination. On the other hand, surface discharges were produced adding traces of carbonized particles on the external surface of a cable termination. Finally, corona discharges were generated adjusting accurately the gap length in a needle-plane electrode configuration where the plane electrode was grounded. 

Furthermore, during the measurement, three types of impulsive noise sources were presented along with the PD pulse sources described above. The first source of impulsive noise was the resonant generator used to perform the HV test, which produces pulses due to the commutation of its IGBTs. The second was caused by a power electronic device connected to the electrical installation of the laboratory, which produces repetitive pulses due to the commutation of its transistors. Finally, random impulsive noise conducted by the grounding conductors, was also measured. 

#### 3.1.2. HV Equipment and Measuring Instrument

**Figure 7:**
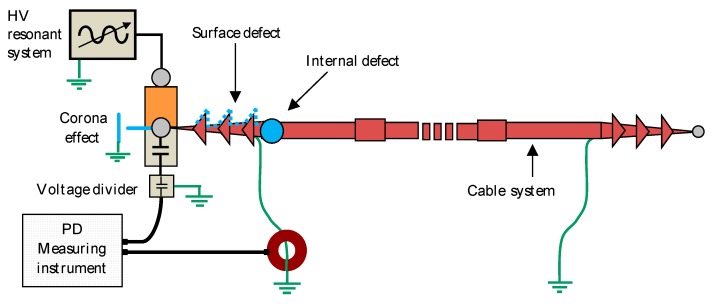
Experimental setup composed by the HV resonant generator, cable system with three defects, HFCT sensor and measuring instrument.

A HV installation was implemented for the measurement of the PD and noise sources, shown in Figure 7. 

The experimental installation was composed of an aluminium 12/20 kV XLPE insulated cable with 240 mm^2^ in diameter and 1500 m in length. The HV source was a 36 kV resonant generator and the test voltage was 15 kV, 50 Hz. A capacitive divider (Figure 7) acquired the reference voltage signal, necessary for the synchronization of the pulses with the test voltage. An HFCT sensor with a bandwidth from 0.2 to 20 MHz was installed in the grounding cable of the termination connected to the HV generator. 

The three PD sources were located together at the cable end connected to the HV generator as it is shown in Figure 7. Usually, in on-line PD measuring or monitoring, the acquisitions are performed in various positions of the facility. The most difficult scenario for the separation of pulses appears when the PD sources are in the same emplacement. It is in that position where the installation of an HFCT sensor is recommended, in order to avoid the effect of the attenuation and distortion of the pulses and to attain more accuracy with the separation technique applied. Thus, in order to evaluate properly the separation method developed, the three PD sources were intentionally placed close to the HFCT sensor, as per Figure 7. It is important to indicate that if the PD sources are not in the same location, the use of previous diagnosis tools that analyse the pulses’ amplitude, polarity, frequency or time of flight enable a first separation of sources usually without many difficulties [27,28].

The pulse-type noise sources were away from the HFCT sensor, as in the majority of real-life measurements performed on-line and in-situ. The source of the IGBTs impulsive noise due to the resonant generator was located at 40 m from the HFCT, the pulse-type noise source due to a power electronic device was at approximately 25 m from the HFCT and the random impulsive noise conducted by the grounding conductors has an unknown origin.

**Figure 8:**
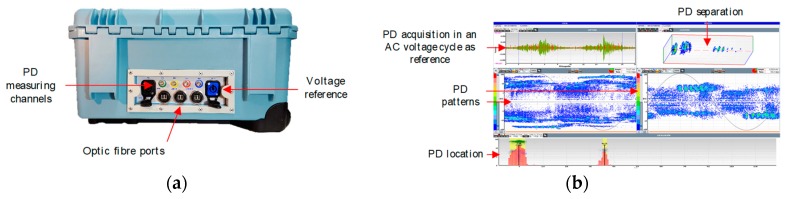
(**a**) Frontal view of the measuring device; (**b**) main interface window.

The measuring instrument used was designed by the researching group of LAT-UPM to perform on-line PD measurements [27]. This instrument is equipped with a data acquisition system with the following specifications: 14 bits of vertical resolution, 50 MHz bandwidth and sampling frequency of 100 MS/s. Figure 8 exhibits a frontal view of the measuring device and the main interface window.

By using the software of this measuring device, continuous noisy signals, present in the background noise, are filtered using a denoising tool based on the wavelet transform [25,27,28]. This enables the selection of signals with impulsive behaviour (as PD pulses) for subsequent processing using the PRPD-time tool developed.

### 3.2. HV PD Measurements

**Figure 9:**
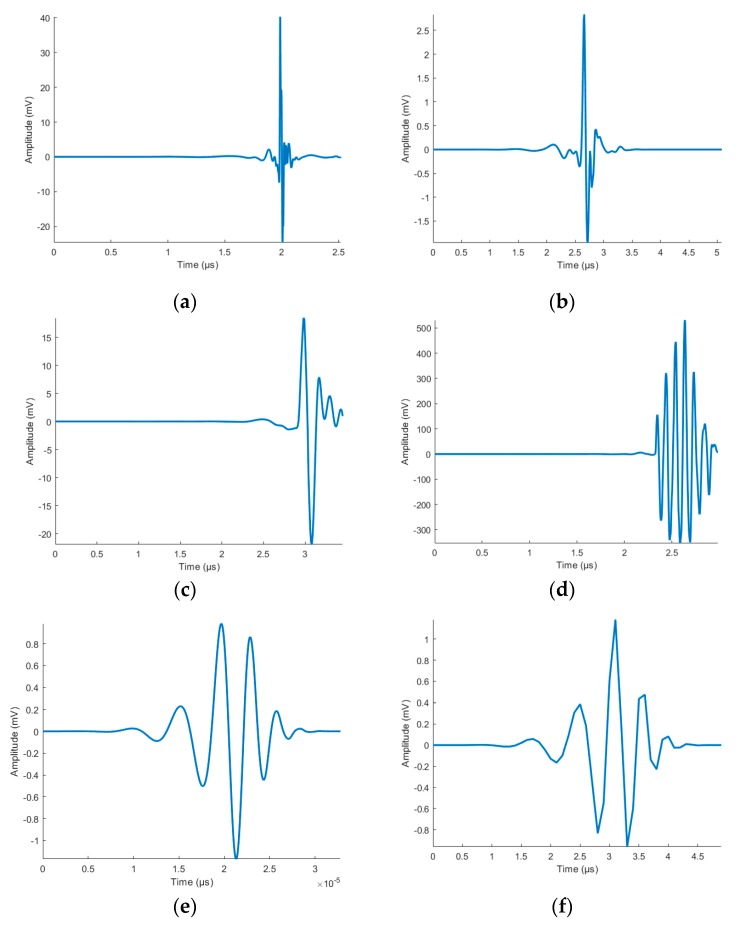
Representative pulses of each signal source measured: corona (**a**); internal PD (**b**); surface PD (**c**); IGBT pulses (**d**); random noise (**e**); and electronic noise (**f**).

The insulation defects placed in the test circuit and the noise sources presented a stable behaviour over time. The sources proposed were previously characterized one by one and the number of pulses for each one is greater than 250 signals. Thus, the number of pulses expected is sufficiently significant to identify, after the separation process, all the pulse sources present in the test circuit. The wave shape of a representative pulse of each source is presented in Figure 9.

**Figure 10:**
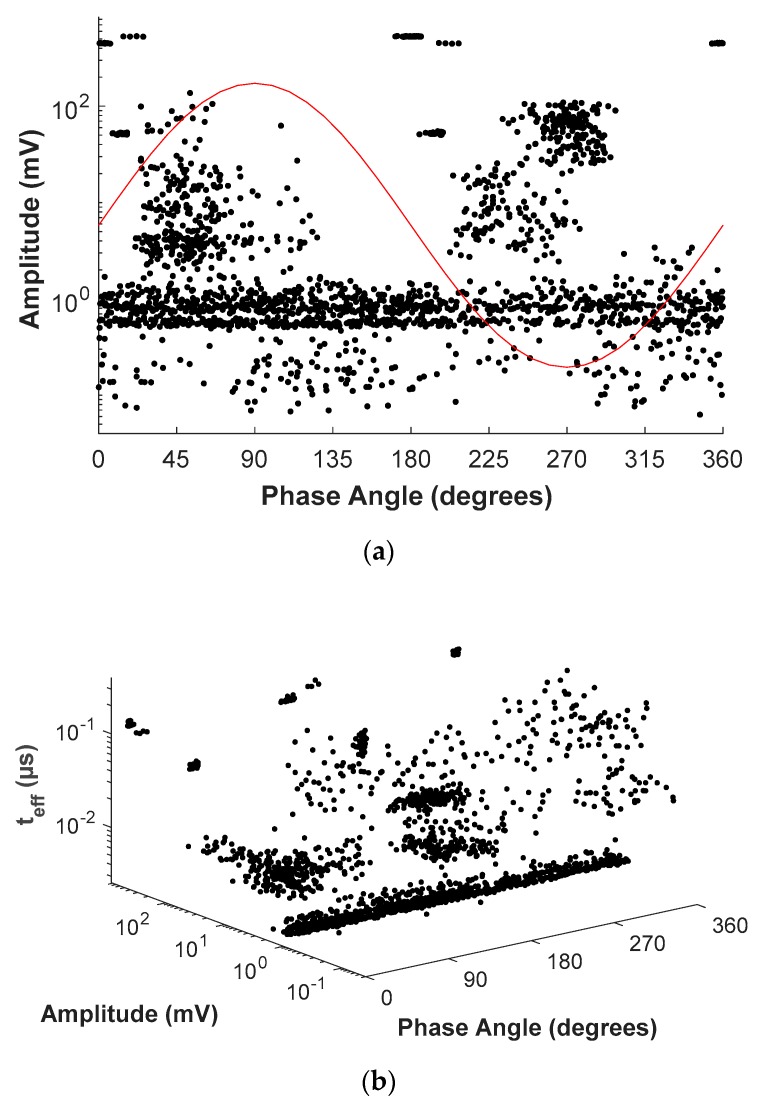
Measured PRPD patterns: (**a**), classical and (**b**), proposed PRPD-*t_eff_* pattern for case study.

The type of sources that were measured simultaneously are listed as follows: corona PD, internal PD, surface PD, electronic noise, pulses from the IGBTs (of the voltage source) and random pulse-shaped noise. Figure 10a shows the measured PRPD and Figure 10b shows PRPD- *t_eff_* patterns obtained in this case study.

As can be seen in Figure 10a, when different PD and impulsive noise sources are measured simultaneously, the differentiation of the involved PD sources and therefore, the realization of an accurate diagnosis of the insulation status of the installation elements is not possible just through the PRPD pattern. 

## 4. Classification Results for the Case Study

**Figure 11:**
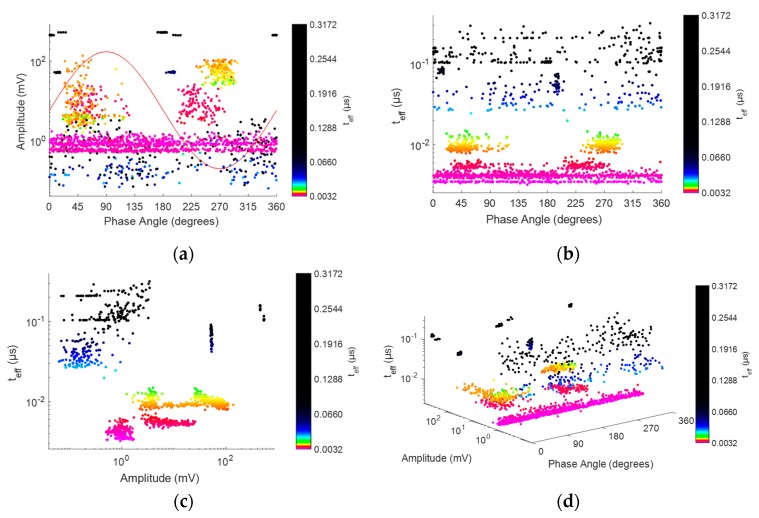
Examples of PRPD-*t_eff_* pattern representations: (**a**) phase angle-amplitude; (**b**), phase angle-*t_eff_*; (**c**) amplitude-*t_eff_* and (**d**) isometric representation of phase angle-amplitude-*t_eff_*.

When the third axis is added, Figure 10b, there is a separation of pulses in clusters related to the sources present in the setup. However, as the differences in the effective time corresponding to different sources are small, the classification process is still difficult. The PRPD-time tool allows overcoming this difficulty due to the logarithmic colouring described in Section 2.3. Figure 11 shows the corresponding PRPD-*t_eff_* pattern for all pulses considered.

Figure 11 shows some of the possible representations of PRPD-*t_eff_* patterns that can be displayed using the sliding list *View* at the lower left-hand side of Figure 6B.

When comparing Figure 10b and Figure 11d it is possible to see that the definition of colours made using the option *Sep. by teff* button allows users to distinguish clusters with different colours and tones depending on the magnitude of *t_eff_*. This clear differentiation among pulses in the PRPD-*t_eff_* pattern in Figure 11d allows users to separate pulses using the containers option controlled by interface in Figure 6A.

By analysing the cluster distribution from the different pairwise representations shown in Figure 11, it is possible to infer the most convenient geometry among the three possibilities by default: parallelepiped, ellipsoid and prism. In addition, the range of effective time values can be determined for defining the tones in order to improve the visualization and separate adequately the clusters in the PRPD-*t_eff_* pattern. For the case corresponding to Figure 11, rectangular containers are convenient because pulses are distributed along all the period and are well separated along the *t_eff_* axis. Table 1, summarizes the parameters selected for containers deducted from Figure 11 and the type of source separated. Table 1 is also presented as a first definition of characteristic regions of the PRPD-*t_eff_* patterns where clusters of pulses, for sources considered in this study, can be found. Other sources as well other measurement scenarios will be considered in future works for a generalization.

**Figure 12:**
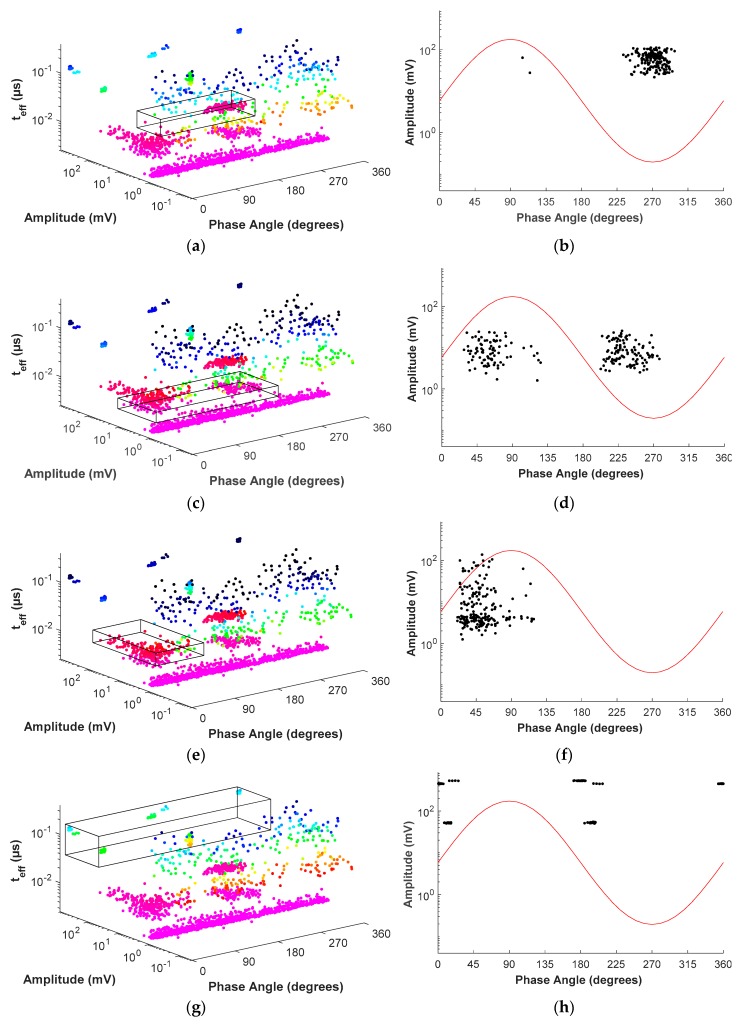

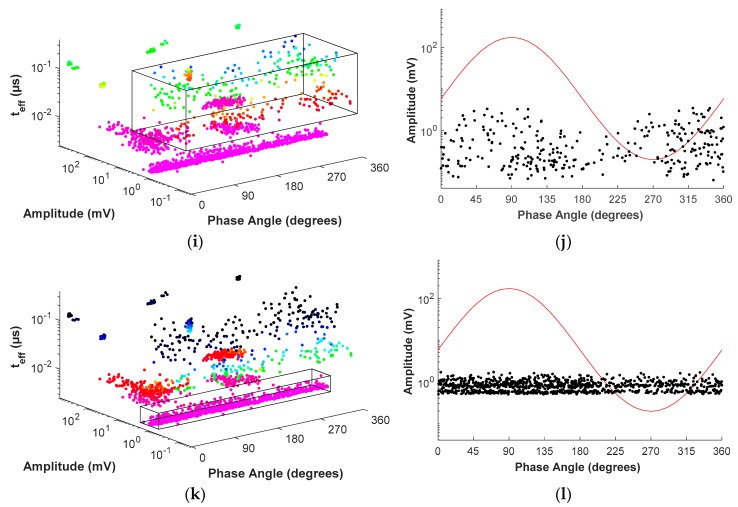
Containers selected superimposed with the PRPD-*t_eff_* pattern and classical PRPD patterns for pulses in each container: (**a**,**b**), corona; (**c**,**d**), internal PD; (**e**,**f**), surface PD; (**g**,**h**), IGBT pulses; (**i**,**j**), random noise; (**k**,**l**), electronic noise.

Figure 12 shows the containers selected superimposed on the PRPD-*t_eff_* patterns for each source. In addition, the PRPD patterns obtained for the PD pulses in containers are shown.

In Figure 12b, it can be seen that the PRPD pattern obtained with pulses separated using the container from Figure 12a exhibits a swarm structure around the maximum amplitude of the negative half cycle with magnitudes dispersed between tens of mV and few hundreds of mV. 

Figure 12d, shows the PRPD pattern for pulses selected with the container of Figure 12c. This PRPD pattern presents pulses in both half cycles. Pulses appear in the ascending period of the positive half cycle and descending period of negative half cycle. The pulses are dispersed among a short range of few tens of mV and few nanoseconds (4–7 ns). 

Figure 12f shows the PRPD pattern corresponding to pulses inside the container of Figure 12e. The PRPD pattern exhibits a structure of pulses dispersed at different magnitudes ranging from zero to few hundred mV at different phases of the positive half cycle, but the higher concentration of pulses can be seen in the ascending period. 

Figure 12h exhibits the PRPD pattern of pulses from the container shown in Figure 12g. The PRPD pattern shows a structure of dashed lines where pulses are well concentrated. They appear in the zero crossings and are in a narrow range of few degrees after the zero crossings.

Figure 12j shows the PRPD pattern for pulses inside the container shown in Figure 12i. This PRPD pattern exhibits a non-correlated cloud of points with the voltage reference and a dispersed low magnitude ranging from zero to few mV. On the other hand, pulses are dispersed in a wide range in the effective time dimension, 18–326 ns.

Figure 12l shows the PRPD pattern corresponding to pulses in the container shown in Figure 12k. The PRPD pattern exhibits a non-correlated behaviour of points with the voltage reference and the pulse magnitudes in a narrow range of few mV.

Taking into account the above analysis and the description of characteristics of PRPD pattern for pulse sources given at the beginning of Section 2, it can be concluded that they correspond, respectively, to the following impulsive sources: corona, internal PD, surface PD, IGBT pulses, random noise and electronic noise.

As can be seen from Figure 12 and Table 1, pulses from different sources have different duration and can be located at different regions of the PRPD-*t_eff_* patterns. It can be seen that corona and surface PD pulses have similar *t_eff_* values and pulse magnitudes, however they can be differentiated by the phase of appearance. In addition, it can be seen that pulses corresponding to internal PD are overlapped in phase and magnitude with corona and surface PD pulses, however they have a different range of effective time that allows their effective separation. 

It can be seen that each PD source was effectively separated and identified. Analysing the individual PRPD patterns obtained, a proper diagnosis of the insulation condition can be performed. For all PD sources considered in this article, parallelepipeds were easily fitted around the clusters. However, other geometrical bodies as described in Section 2.3 can be used. 

## 5. Conclusions

A novel and effective method for impulsive sources separation was proposed, which considers the effective time as a feature parameter along with the pulses’ amplitudes and the phase in which they are acting. In addition, in order to separate sources of pulses by clustering using the effective time parameter, a powerful and interactive interface tool was implemented in a Matlab application named PRPD-time. The method and the Matlab application were validated using experimental measurements considering six different impulsive sources acting simultaneously.

In the PRPD-time tool, the successful source pulse separation is implemented using two complementary tools. The first, allows including the effective time as a feature parameter and PRPD-*t_eff_* patterns can be obtained with a dynamical colouring process that enhances the visualization of clusters. The second allows separating the clusters previously differentiated and analysing their PRPD pattern for identifying the corresponding impulsive source.

PRPD-time tool is a very interactive application that requires low-computational capabilities and gives competitive advantages over other methods and technologies present in the current market. The proposed method is very easy to implement, considers feature parameters with physical meaning and does not require previous training or expert knowledge.

## Figures and Tables

**Table 1:** Containers parameters for each PD source.

Source.	X (degree)		Y (mV)		Z (μs)	
	Min	Max	Min	Max	Min	Max
Corona	104.4	302.4	19.18	115.2	0.007	0.015
Internal PD	25.2	280.8	1.591	28.57	0.004	0.007
Surface PD	18	118.8	1.18	140.6	0.007	0.014
IGBT pulses	0	360	47.02	567.1	0.039	0.16
Random noise	0	360	0.059	3.529	0.018	0.326
Electronic noise	0	360	0.481	1.941	0.003	0.007
